# Echoes of the Month

**Published:** 1923-04

**Authors:** 


### ECHOES OF THE MONTH.

### END OF THE PARIS MORGUE.

The Paris Morgue is no more. It is to be replaced by an entirely new type of institution. The original Morgue, which overhangs the riverside close by Notre Dame, was founded in 1864. It was devoted to the reception of the bodies of those to whom death had come by other than natural means, and the bodies of people who had met with violent ends were in many cases preserved there for many years until the mystery of their deaths could be solved. Little by little, however, the Morgue was transformed into something more, and from 1877 it had been the headquarters of the School of Legal Medicine. For this purpose, in view of the rapid progress of scientific research, it soon became seriously inadequate, and, in 1908 a project for the establishment of a definite medico-legal institute, of which the old Morgue should form part, was definitely put forward. This project has now been carried out. The new institution includes laboratories for the study of legal medicine, toxicology and judicial identification, as well as three commodious rooms for performing post-mortem examinations. The premises also include a criminological museum.

### CIVILISATION AS A CAUSE OF DISEASE.

Sir William Arbuthnot Lane, lecturing for The People's League of Health, on "The Sewage System of the Human Body," called attention to the fact that civilisation is purchased very dearly by the development of a large number of chronic diseases from which natives living in a low state of civilisation are quite free. He showed that in the huge populations inhabiting the Himalayas, indigestion, appendicitis, gastric and duodenal ulcer, colitis and cancer are absolutely unknown and that among the native Indians and mixed bloods in South America cancer does not exist. He pointed out that when the negro acquires the habits and surroundings of civilisation, as in the United States, he is affected by cancer and the above-mentioned diseases just as frequently as the white man. Therefore, it is obvious that if the white man leads the same life and eats the same food as the native, he will not only escape those diseases, but also the innumerable affections of the lungs, kidneys, heart, joints, teeth and other [structures that attack the civilised man because of the lowering of the vitality of his tissues and of their resisting power to organisms consequent on their being poisoned by contaminated food products obtained from a foul intestine.

### MONTAIGNE FOR LONGEVITY.

Dr. Armaingaud, of the French Academy of Medicine, has revealed to us the secret of longevity. His secret does not involve any experiments with our glands; it is much simpler —it consists in reading Montaigne. Dr. Armaingaud, it appears, took his first dip into the essays when he was nine years old, and he is still reading them now that he is 81.

### THE PRIEST AS MEDICINE MAN.

After acknowledging the value of the ministrations of the clergy during prolonged sickness and periods of crisis, Dr. George M. Robertson, in his annual report as physician-superintendent of the Royal Hospital at Morningside, Edinburgh, remarks that "there exists a temptation at such times which a few clergymen appear unable to resist, of absorbing the professional sphere and the functions of the physician. These persons, though uneducated in medical science, which involves the most laborious training connected with any of the learned professions, pose as healers. . . They cure by the possession of a mysterious 'gift of healing,' or by something not essentially different from the performance of a miracle, the initiation of which is their peculiar prerogative. No one limits the possibilities of spiritual or divine power, and the cure of all diseases, by whatever means, is through divine agency, But," Dr. Robertson adds, "the laws of space and of gravitation are no more an obstacle to this power than are those of anatomy and physiology, for mountains may be removed, yet we do not hear of the clergy initiating miracles in this sphere or performing feats of an engineering kind. When the foundations of the pillars supporting the dome of a great church subside, or the spire of some ancient cathedral shows cracks, they open subscription lists and call in the services of an architect, yet they rush in to restore a temple not made with hands."

### VACCINATION 2,000 YEARS OLD.

"Vaccination is an outgrowth of man's effort to protect himself from pestilence by using nature's methods of defence," says Dr. G. W. McCoy, director of the Hygienic Laboratory of the United States Public Health Service. "Primitive man noticed that recovery from a first attack by most diseases gave immunity against other attacks; and some 2,000 years ago he began to inoculate his fellows with smallpox when conditions seemed propitious instead of waiting for nature to do it at some time when conditions might be very unpropitious. Inoculations against smallpox were made in India and in China as early as 300 B.C. Later, when the disease reached Europe, inoculation went with it, supplemented by a new method called 'selling smallpox'—exposing a well person to contact with one ill with the disease, so that if he survived he would be proof against it. Inoculation differs somewhat from vaccination as devised by Jenner, but the principle is the same. Moreover, long before Jenner's day it was known that an attack of cowpox gave immunity from smallpox; and records show that men who had recovered from cowpox had themselves inoculated with smallpox to make the proof conclusive. Jenner, however, as he himself says, 'placed vaccination on a rock,' where he knew it would be immovable."

### GIFTS OF BLOOD.

The Bradford Royal Infirmary is advertising for persons willing, if found suitable, to make gifts of blood when these are required for transfusion purposes. The object is to have at the Infirmary a list of persons who can be called upon to meet sudden needs. Transfusion is an operation with a long history, but, although its use has been fairly common for some time in America, only a few English surgeons employed it at all frequently here until the war revealed its full possibilities, and also emphasised the need for care in choosing suitable donors. During the war the technique of transfusion was much developed; direct transfusion from vein to vein largely fell out of use, and blood was carried from donor to recipient either in a specially prepared glass tube or in a syringe. This method is, in practice, much simpler, and enables the precise quantity of blood transfused to be measured far more accurately. In 1918, during the heavy spring fighting, a further method was evolved of drawing blood off from suitable donors, and preserving it ready for injection under conditions preventing coagulation. Men were always found willing to make gifts of blood, and when an order was issued enjoining three weeks' leave in England for blood donors, the operation even came to be regarded by lightly-wounded men as a coveted privilege.

### THE TREATMENT OF LUPUS.

In a lecture at the London Hospital, Dr. Sequeira spoke of the value of Light Baths in the treatment of lupus. He said that last summer he decided to try exposing cases of lupus which failed to be influenced by the local application of concentrated light (the Finsen light) to light baths, and in August a 25-ampere arc lamp was installed. Immediate improvement was observed. Then a 50-ampere arc lamp was obtained. The carbon-arc light is placed about three feet from the ground. The patients, clad only in bathing drawers, are seated around this. The whole of the body and the limbs are exposed to the light of the arc, care being taken to protect the eyes by thick shields when the anterior surface is under treatment. At first the sittings are of half-an-hour's duration, and they are gradually extended until both anterior and posterior surfaces are exposed for two hours. The effects observed are rapid darkening of the skin ("sunburn"), rapid healing of the disease, increase in body weight, and improvement in the general condition, listless lads becoming bright and keen on their cures. There is also in some cases an increase in the number of white blood corpuscles The light bath will not displace the Finsen light, but is of "the highest value in improving the general nutrition."

